# Narrowband UVB treatment induces expression of WNT7B, WNT10B and TCF7L2 in psoriasis skin

**DOI:** 10.1007/s00403-019-01931-y

**Published:** 2019-05-14

**Authors:** Malin Assarsson, Jan Söderman, Albert Duvetorp, Ulrich Mrowietz, Marita Skarstedt, Oliver Seifert

**Affiliations:** 1 Division of Dermatology and Venereology, Ryhov Hospital, Region Jönköping County, 55185 Jönköping, Sweden; 2 Division of Medical Diagnostics, Ryhov Hospital, Region Jönköping County, Jönköping, Sweden; 3 Division of Dermatology and Venereology, Skånes University Hospital, Malmö, Sweden; 4 Psoriasis-Center, Department of Dermatology, University Medical Center Schleswig-Holstein, Campus Kiel, Kiel, Germany; 5 Department of Clinical and Experimental Medicine, Faculty of Medicine and Health Sciences, Linköping University, Linköping, Sweden

**Keywords:** WNT/β-catenin signaling, WNT proteins, Inflammation, Gene expression, Single nucleotide polymorphisms

## Abstract

WNT/β-catenin signaling pathways play a pivotal role in the human immune defense against infections and in chronic inflammatory conditions as psoriasis. Wnt gene alterations are linked to known comorbidities of psoriasis as obesity, diabetes and Crohn’s disease. The objective of this study was to investigate WNT7B, WNT10B, WNT16 and TCF7L2 gene and protein expression in lesional and non-lesional skin and in the peripheral blood of patients with chronic plaque psoriasis compared with healthy individuals. To investigate the effect of narrowband UVB radiation, expression of these genes were analyzed before and after narrowband UVB treatment. Associations between single nucleotide polymorphisms for WNT7B, WNT10B, WNT16 and TCF7L2 genes and psoriasis were tested. Our results show significantly decreased WNT7B, WNT10B and TCF7L2 gene expression in lesional skin compared with non-lesional skin and healthy controls. Narrowband UVB treatment significantly increased expression of these genes in lesional skin. Immunohistochemistry shows increased WNT16 expression in lesional skin. No significant differences in allele or genotype frequencies for Wnt or TCF7L2 gene polymorphisms were found between patient and control group. This study shows for the first time significant UVB induced upregulation of WNT7B, WNT10B and TCF7L2 in patients with psoriasis and suggests a potential role of these genes in psoriasis pathogenesis.

**Electronic supplementary material:**

The online version of this article (10.1007/s00403-019-01931-y) contains supplementary material, which is available to authorized users.

## Introduction

WNT proteins are a family of secreted glycoproteins with 19 isoforms. Recent reports suggest a role of WNTs in inflammation, psoriasis and the human immune defense against infections [6, 22, 23, 37, 40, 42]. WNTs can activate two distinct signaling pathways: the canonical WNT/β-catenin pathway and the β-catenin-independent, non-canonical pathway [9].

Psoriasis is a chronic systemic inflammatory disease characterized by activation of both innate and adaptive immunity [34]. Dendritic cells play a key role in linking innate and adaptive immunity and in balancing inflammatory and regulatory responses. Interestingly, a recent study showed that activation of WNT/β-catenin signaling in dendritic cells is critical for promoting tolerance and limiting inflammation [45].

In spite of the important roles WNT proteins play in cell proliferation and differentiation and their role in innate immunity, not much is known about the expression and the potential function of WNT isoforms under pathophysiological conditions, such as in psoriasis. WNT16 plays a role in mediating keratinocyte proliferation [46], and one study shows lower WNT7B gene expression in lesional skin compared to non-lesional skin while WNT16 expression was unchanged [43]. Two reports describe increased expression of WNT5A mRNA and protein in lesional skin of patients with psoriasis [22, 43], and interestingly, recent data suggest WNT5a to be a link between psoriasis, obesity and metabolic complications [17, 33]. The transcription factor 7-like 2 (TCF7L2) gene encodes a transcription factor involved in the WNT signaling pathway, and numerous studies show a correlation between TCF7L2 single nucleotide polymorphisms (SNPs) and dyslipidemia, metabolic syndrome and type 2 diabetes, [19, 41] which are known associated diseases of psoriasis [20].

The aim of this study was to describe the expression of WNT7B, WNT10B, WNT16 and TCF7L2 in lesional and non-lesional skin and in whole blood of patients with psoriasis compared to healthy individuals and to investigate if SNPs in these genes can be correlated to psoriasis. Previous studies show that ultraviolet (UV) radiation upregulates members of the WNT/β-catenin pathway [16, 50]. To investigate the effect of UV radiation on WNT7B, WNT10B, WNT16 and TCF7L2, gene expression levels were analyzed before and after narrowband UVB (nbUVB) treatment.

## Materials and methods

### Study subjects

**Fig. 1:**
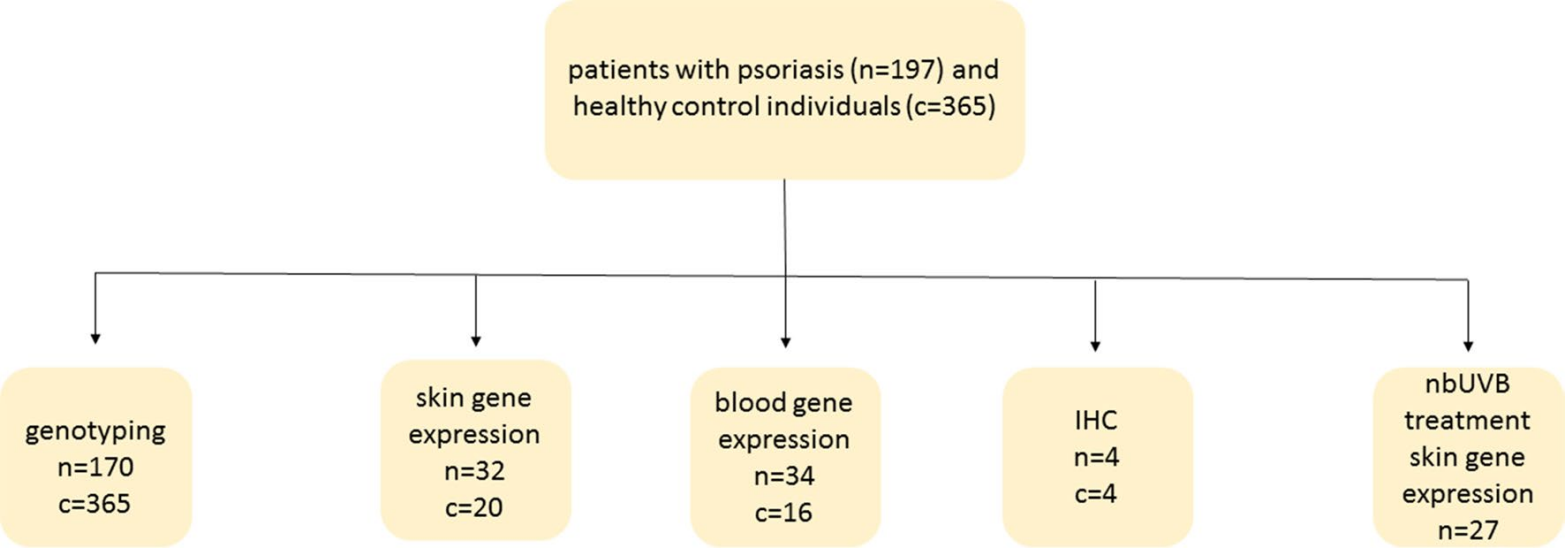
Illustration of patient flow and number of patients with chronic plaque psoriasis (*n*) and healthy control individuals (*c*) included in the study and the different WNT7B, WNT10B, WNT16 and TCF7L2 analysis performed (*nb* narrowband, *IHC* immunohistochemistry)

Patients with chronic plaque psoriasis were recruited from the outpatient clinic at the Division of Dermatology, Ryhov Hospital, Jönköping, Sweden. Healthy controls were recruited from patients visiting the same clinic for evaluation of benign nevi. Whole blood samples for genotyping were obtained from these controls and from blood donors from Ryhov Hospital, Jönköping, Sweden. Participants’ demographic data are summarized in Table 1. The type of analysis performed and the number of individuals included for each analysis are illustrated in Fig. 1. Study subjects did not use any systemic or topical anti-psoriatic treatments 2 weeks prior to study inclusion. This study was conducted in compliance with good clinical practice and according to the Declaration of Helsinki Principles. Written informed consent was obtained from all subjects under protocols approved by the Local Ethics Committee, Linköping University, Sweden. Sex, age and body mass index (BMI) were recorded for all individuals and an assessment of disease severity using the Psoriasis Area and Severity Index (PASI) was recorded for subjects with psoriasis.

**Table 1:** Study population’s demographic data, psoriasis area and severity index (PASI), body mass index (BMI) and age shown as mean and standard deviation (SD)

	Psoriasis	Control
Genotyping		
Study subjects (*n* female, % female)	170 (61, 36%)	365 (201, 55%)
Age	51.7 (15.8)	55.4 (10.5)
Skin gene expression		
Study subjects (*n* female, % female)	32 (12, 38%)	20 (13, 65%)
Age	54.8 (13.9)	45.7 (14.3)
PASI	7.2 (6.5)	–
BMI	27.5 (3.5)	23.9 (1.7)
Immunohistochemistry		
Study subjects (*n* female, % female)	4 (2, 50%)	4 (2, 50%)
Age	62.0 (12.1)	43.3 (10.8)
PASI	6.7 (3.7)	–
Blood gene expression		
Study subjects (*n* female, % female)	34 (15, 44%)	16 (9, 57%)
Age	57.6 (14.8)	42.5 (12.0)
PASI	6.4 (5.5)	–
BMI	27.8 (4.5)	24.4 (2.0)
Gene expression nbUVB treatment		
Study subjects (*n* female, % female)	27 (7, 21%)	–
Age	48.3 (15.1)	–
PASI before	7.9 (4.5)	–
PASI after	2.1 (1.8)	–
BMI	26.5 (4.1)	–

### Sampling methods

Full-thickness punch biopsies for WNT7B, WNT10B, WNT16 and TCF7L2 gene expression and immunohistochemistry (IHC) were taken from non-lesional skin (4 mm in diameter; at least 10 cm distance from any psoriatic lesion) and from the active margin of a psoriatic plaque (4 mm diameter) after application of local anesthetics. In healthy controls, biopsies were obtained from corresponding anatomical sites. Immediately upon removal, biopsies were stored in either formalin for IHC or RNA*later*, RNA Stabilization Reagent (Qiagen, Hilden, Germany) for gene expression analysis and stored at − 150 °C.

Whole blood samples were taken from patients with psoriasis and controls for WNT7b, WNT10b, WNT16 and TCF7L2 gene expression and genotyping. For genotyping venous blood samples were collected in BD Vacutainer EDTA tubes (BD Biosciences). The tubes were centrifuged at 2000×*g* for 10 min and the buffy coat was frozen in − 80 °C for subsequent DNA extraction and genotyping. Tempus™ Blood RNA tubes (Thermo Fisher Scientific Inc) were frozen in − 80 °C for subsequent RNA extraction and gene expression.

For analysis of WNT7B, WNT10B, WNT16 and TCF7L2 gene expression in response to nbUVB treatment, full-thickness 2 mm punch biopsies were taken after application of local anesthetics from a single psoriatic plaque and from non-lesional skin at least 10 cm distance from any psoriatic lesion of patients before and after receiving a full nbUVB treatment series according to standard clinical protocol at the Division of Dermatology, Ryhov Hospital. The location of the biopsies before treatment was recorded to ensure that biopsies after treatment were taken from approximately the same location.

### Phototherapy protocol

NbUVB (311 nm) therapy was administered using a Waldmann 7002 cabin (Waldmann Medizintechnik, Villingen-Schwenningen, Germany). The patients were treated on average 2.3 times (± 0.7) per week and the mean treatment period was 10.4 weeks (± 3.6). The mean maximum dose reached was 2.64 J/cm^2^ (± 1.2) at the end of the treatment period. Energy output was measured with a standard intrinsic UV meter. Initial dose was dependent on skin phototype. If the initial dose was tolerated, the previous dose was increased by 20% at each visit. When a previous treatment resulted in erythema, no treatment was given the next day or the dose was decreased, depending on whether the erythema was asymptomatic or severe and painful.

### Immunohistochemistry

WNT7B, WNT10B, WNT16 and TCF7L2 staining was performed using a standard protocol on 4 µm sections from formalin-fixed paraffin-embedded tissue blocks as previously described [12]. Sections were subsequently incubated for 30 min at a concentration of 1:1000 with a primary rabbit anti-human WNT7B antibody (1.0 mg/mL, Sigma Aldrich, St Louis, USA), with primary mouse anti-human WNT10B anti-body at a concentration of 1:50 (0.5 mg/mL, R&D, Minneapolis, USA), with primary rabbit anti-human WNT16 anti-body at a concentration of 1:40 (0.1 mg/mL, Sigma Aldrich, St Louis, USA) and with primary mouse anti-human TCF7L2 antibody at a concentration of 1:200 (1.0 mg/mL, Sigma Aldrich, St Louis, USA).

Detection of primary antibodies was performed using the MACH4 Universal HRP-Polymer Detection System (Biocare Medical, Concord, USA) in the IntelliPATH FLX system (Biocare Medical, Concord, USA) as previously described [32]. Sections were counterstained with Hematoxylin and rehydrated before coverslips were added. Microscopy of the sections was performed using a Zeiss light microscope (Carl Zeiss Microscopy GmbH, Göttingen, Germany) along with the Zen lite software (Zeiss).

Positive control was performed with colon tissue known to express high levels of TCF7L2 and tissue from pharyngeal tonsils, appendix and liver for WNT7B, WNT10B and WNT16.

### RNA extraction, cDNA synthesis and RNA quantification

Total RNA was purified according to the manufacturer´s instructions. Briefly, biopsies were homogenized using a TissueRuptor and disposable probes (Qiagen, Hilden, Germany), and RNA was purified using the RNeasy Fibrous Tissues mini kit (Qiagen). RNA from stabilized blood was purified using the Tempus Spin RNA Isolation Reagent kit (Life technologies). Concentration and purity was measured using a Nanodrop ND-1000 (Thermo Fisher Scientific Inc., Waltham, USA), and RNA integrity was assessed using the RNA integrity number with a 2100 Bioanalyzer (Agilent technologies, Santa Clara, USA) and RNA was stored at − 80 °C.

RNA was reverse transcribed using the High capacity cDNA reverse transcription kit with RNase inhibitor (Applied Biosystems, Waltham, USA), according to the manufacturer’s instructions, and the resulting cDNA was stored at − 80 °C.

Gene expression was analyzed on the 7500 Fast real-time PCR system (Applied Biosystems) and the standard run mode using TaqMan Universal Master Mix no UNG (Applied Biosystems) and TaqMan Gene Expression Assays (Applied Biosystems) (Supplementary Table 1). For each assay and sample, cDNA based on 10 ng total RNA were analyzed in a total volume of 20 μL.

Threshold cycle (c_t_) values were established using the 7500 software version 2.0.6 (Applied Biosystems). Reference genes (TBP, ACTB and GAPDH) were evaluated for low sample-to-sample variation using the NormFinder [3] algorithm implemented in the GenEx Professional software version 5.4.2.128 (MultiD Analyses AB, Gothenburg, Sweden). WNT and TCF7L2 *c*_t_ values were normalized to the reference gene showing the best stability value (GAPDH reference gene for the nbUVB treatment population and the blood samples and ACTB for skin gene expression). Relative gene expression was compared using the comparative Ct (2^−ΔΔct^) method [35].

### DNA extraction and genotype determination

DNA was extracted from the buffy coat using the QiaAmp DNA blood kit (Qiagen). 10 ng of each DNA sample was genotyped using the TaqMan Universal PCR Master mix II (Applied Biosystems), TaqMan SNP genotyping assays (Supplementary Table 2) and the CFX96 Real-time System, C1000 Touch Thermal Cycler (Bio-Rad Laboratories Inc., Hercules, USA). SNP marks with a minor allele frequency of 0.2 and a pairwise correlation (*r*^2^) of 0.8 were selected using the Haploview software version 4.2 (available at http://www.broadinstitute.org/haploview/haploview) and genotype data based on individuals of European descent available from the 1000 genomes project (http://www.internationalgenome.org).

### Statistical analysis

Statistical evaluation of multiple groups was performed by Kruskal–Wallis ANOVA by Ranks and comparisons of mean ranks of all pairs of groups [44] were performed as a post hoc test. To account for multiple comparisons, *p* values were adjusted according to Bonferroni. Wilcoxon signed rank test was used when analyzing dependent samples (i.e. effect of nbUVB treatment on WNT7b, WNT10B, WNT16 and TCF7L2 expression) with Bonferroni correction for multiple testing. Mann–Whitney test was performed for evaluation of two groups. Hardy–Weinberg equilibrium was confirmed for the investigated genotypes using the exact test implemented in the Haploview software version 4.2 [4]. Chi-Square test was used to investigate if there is a correlation between tested SNPs and psoriasis, and correction for multiple testing was performed using the Permutation test (1000 permutations) in the Haploview software. All statistical analysis, with exceptions stated above, was performed using Statistica 12 software (Statistica, Tulsa, USA) and SPSS statistics version 22 (IBM, New York, USA). Bonferroni corrected *p* values were obtained by multiplying each uncorrected *p* value by the number of tests, and corrected *p* values < 0.05 were considered significant.

## Results

### Decreased WNT7B, WNT10B and TCF7L2 gene expression in lesional skin of patients with psoriasis

**Fig. 2:**
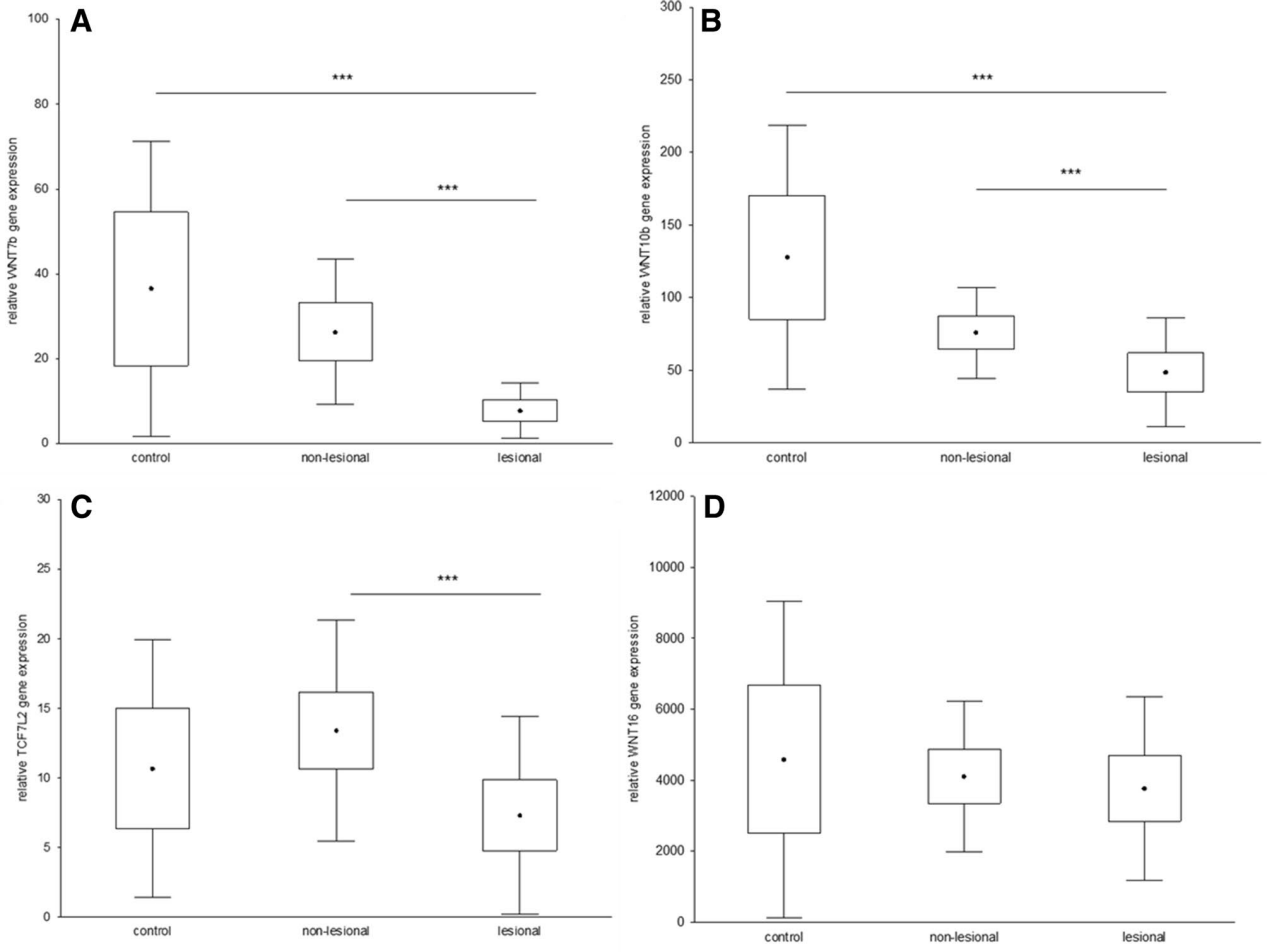
Significantly decreased WNT7B (**a**), WNT10B (**b**) and TCF7L2 (**c**) gene expression in lesional compared with non-lesional skin in patients with psoriasis and significantly decreased WNT7B (**a**) and WNT10B (**b**) in lesional skin of patients with psoriasis compared with healthy controls (control *n* = 20, non-lesional and lesional *n* = 32, ^•^mean, box = mean ± confidence interval, whiskers = mean ± SD, ***p < 0.001)

Gene expression analysis revealed significantly decreased WNT7B, WNT10B and TCF7L2 expression levels in lesional skin compared to non-lesional skin of patients with psoriasis (*p* < 0.001) (Fig. 2a–c). WNT7B and 10B gene expression levels were also significantly decreased in lesional skin compared to skin from control individuals (*p* < 0.001) (Fig. 2b, c). There was no significant difference in WNT16 gene expression between lesional skin, non-lesional skin and skin from control individuals (Fig. 2d). There was no correlation between PASI and gene expression in any of the studied genes.

### UVB treatment induces WNT7B, WNT10B and TCF7L2 gene expression in lesional skin of patients with psoriasis

**Fig. 3:**
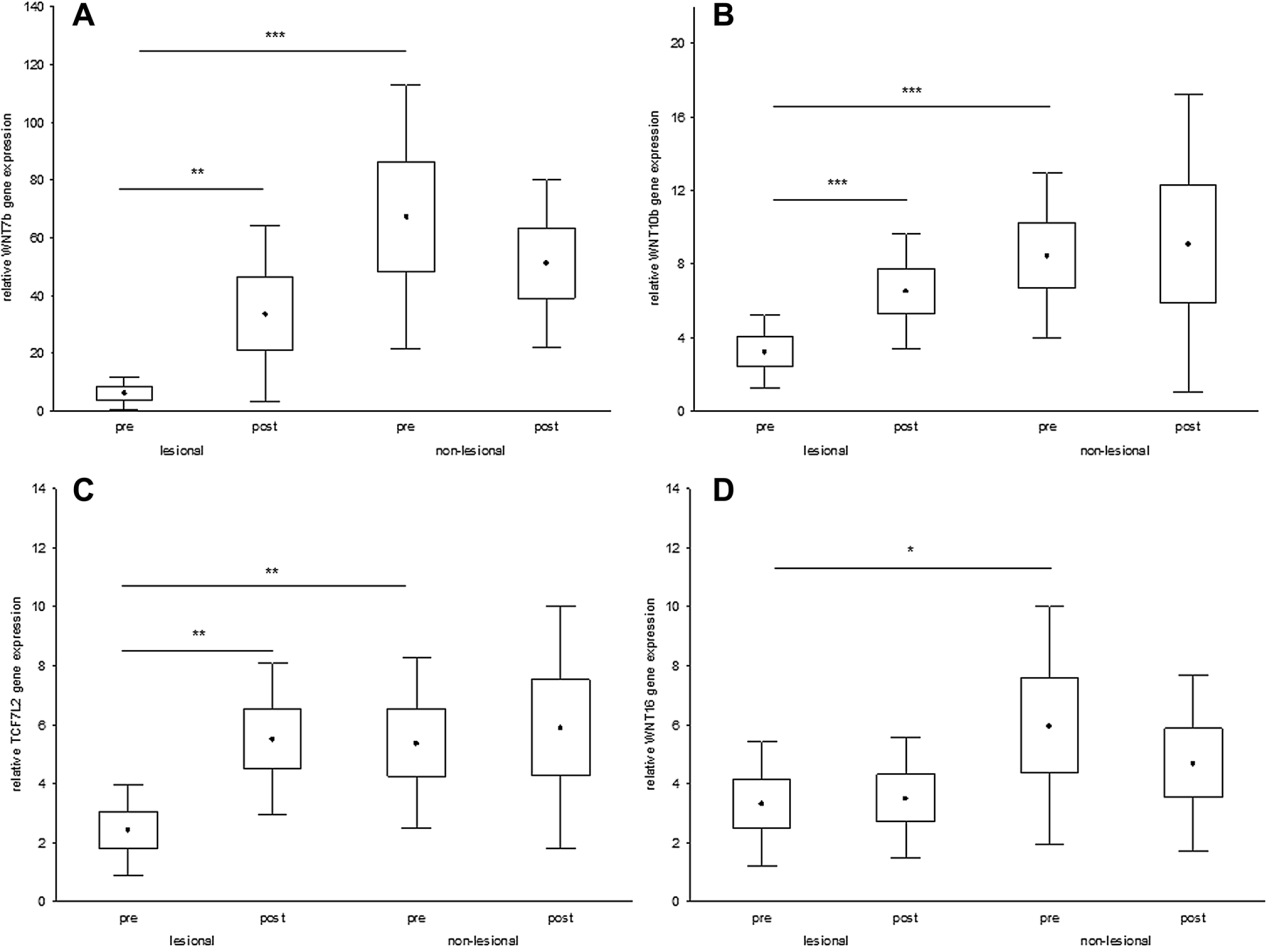
Narrowband UVB treatment significantly induces WNT7B (**a**), WNT10B (**b**) and TCF7L2 (**c**) gene expression in lesional skin of patients with psoriasis (pre = before and post = after nbUVB treatment, *n* = 27, ^•^mean, box = mean ± 0.95 confidence interval, whiskers = mean ± SD, *p < 0.05, **p < 0.01 and ***p < 0.001)

Treatment with nbUVB induced a significant increase of WNT7B (*p *< 0.01, Fig. 3a), WNT10B (*p* < 0.001, Fig. 3b) and TCF7L2 (*p* < 0.01, Fig. 3c) gene expression in lesional skin of patients with psoriasis. The mean number of nbUVB treatment sessions performed was 21 [± 4.3 standard deviation (SD)] and mean PASI improvement was 70% (± 23% SD). There was no correlation between PASI and gene expression in any of the studied genes before or after nbUVB treatment.

### WNT7B, WNT10B, WNT16 and TCF7L2 gene expression in peripheral blood from patients with psoriasis

Levels of WNT7B, WNT10B, WNT16 and TCF7L2 gene expression in peripheral blood samples of patients with psoriasis were not significantly different compared to healthy individuals.

### WNT7B, WNT10B, WNT16 and TCF7L2 protein expression in skin from patients with psoriasis and from healthy controls

**Fig. 4:**
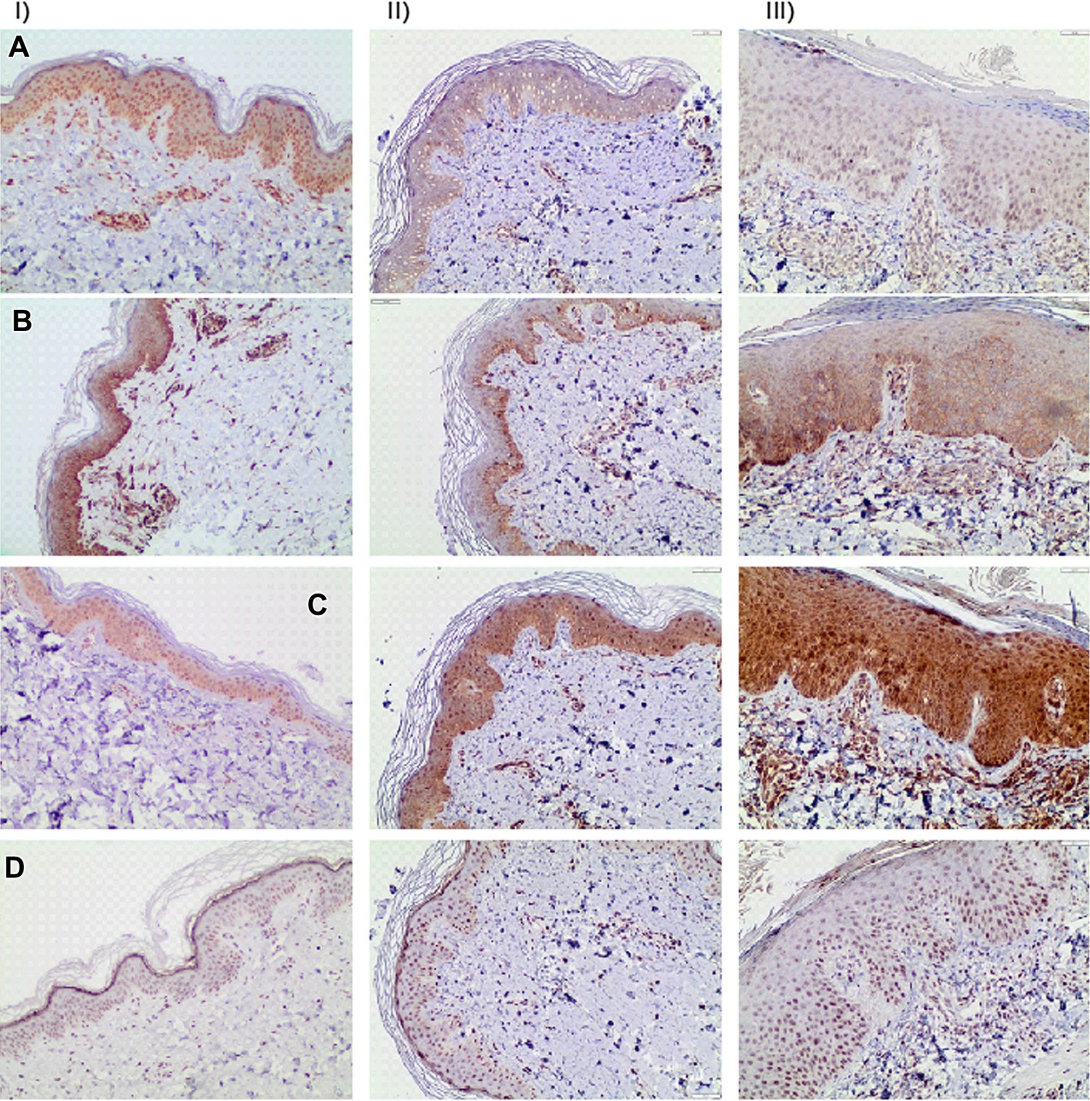
Expression of WNT7B (**a**), WNT10B (**b**), WNT16 (**c**) and TCF7L2 (**d**) protein in skin from healthy controls (I), in non-lesional (II) and lesional skin (III) from patients with psoriasis analyzed by immunohistochemistry. Histology from one representative patient is shown (hematoxylin, *n* = 4, *c* = 4, 20 × magnification)

WNT7B protein expression was more prominent in skin from healthy control subjects compared to non-lesional and lesional skin from patients with psoriasis (Fig. 4a). WNT10B is suggestively slightly more prominent in skin from healthy control subjects compared to non-lesional and lesional skin (Fig. 4b). A more intense expression of WNT16 was observed in lesional skin from patients with psoriasis compared to healthy control subjects and non-lesional skin (Fig. 4c). Expression of TCF7L2 appeared to be similar in skin from healthy control subjects compared to non-lesional and lesional skin from patients with psoriasis (Fig. 4d).

### No association between gene polymorphisms and psoriasis

Of the analyzed SNPs for WNT7B, WNT10B, WNT16 and TCF7L2, no significant association to psoriasis was found. Risk allele A in WNT10B SNP rs1051886 was significant when uncorrected for multiple testing (Supplementary Table 3). Neither the patient nor the control group showed any significant deviation in genotypic frequency as assessed by the Hardy–Weinberg equilibrium.

## Discussion

In spite of the well-known role of WNT signaling in inflammatory processes and in the regulation of stem cell proliferation and differentiation, they have so far only marginally been analyzed in psoriasis. Of the WNT proteins, WNT5a is the one most extensively studied in psoriasis and has been suggested to have a role in inducing the marked vascular changes, epidermal proliferation, and amplification of inflammatory responses seen in lesional skin [22, 51].

Our study shows significantly decreased gene expression of WNT7B, WNT10B and TCF7L2 in lesional skin of psoriatic patients compared to non-lesional skin. WNT7B and WNT10B are also significantly decreased in lesional skin compared to healthy controls. Interestingly, our data show for the first time that gene expression of WNT7B, WNT10B and TCF7L2 all significantly increase in lesional skin after nbUVB treatment.

Previous studies suggest that WNT10B regulates hematopoietic, mammary, and mesenchymal stem cells and seems to play a diverse role in several diseases including breast cancer, obesity and osteoporosis [49]. In the skin, WNT10B has been reported to induce hair follicle regeneration [52] and has been linked to systemic sclerosis [48]. To our knowledge, there is no current data on the expression of WNT10B in psoriasis. However, osteopenia and osteoporosis have been associated with psoriasis and WNT10B signaling appears to impact bone formation [24]. In mouse models, expression of WNT10B led to an increase in bone mass and strength [36] and the deletion of WNT10B in mice resulted in 30% reduction of bone volume and bone mineral density [5]. Serum IL-17A levels inversely correlate with bone volume and bone mineral density and IL-17A inhibits WNT signaling in osteoblasts and osteocytes. This led to the assumption that upregulation of IL-17A in psoriasis inhibits WNT signaling in osteoblasts and osteocytes, thereby reducing bone formation rate [47]. Yet, these results are disputed since some studies do not show an increased risk of osteoporosis in patients with psoriasis [27, 28, 38]. IL-17A produced in keratinocytes might be responsible for the low WNT expression in lesional skin found in our study. It is interesting to speculate that chronic skin inflammation affects WNT expression and hence the regulation of keratinocyte proliferation. In this context, the anti-inflammatory effect of nbUVB treatment might reestablish regular WNT secretion and epidermal proliferation. Our IHC results on WNT10B protein levels in the skin suggest a slightly more prominent protein expression in controls compared to lesional and non-lesional skin of patients with psoriasis. However, because of the uncertainty in quantifying proteins using IHC and the low number of participants, further studies are needed to draw any certain conclusions from this.

In line with our results, WNT7B gene expression has previously been found to be lower in lesional skin of patients with psoriasis compared to non-lesional skin [22, 43]. The IHC results in our study also suggest decreased WNT7B expression in lesional and non-lesional skin compared to control skin. Several previous studies have found that the canonical WNT pathway is suppressed in lesional skin of psoriatic patients [22, 43]. It can be speculated that this suppression is linked to the decreased gene expression of WNT7B and WNT10B found in lesional skin in our study since WNT7B has been shown to potentiate the activity of canonical WNTs [2] and upregulation of WNT10B has been shown to activate canonical WNT signaling [25].

We found no significant difference in the gene expression of WNT16 when comparing lesional to non-lesional skin of psoriatic patients or compared to healthy controls, which is in concordance with previous studies [43]. However, we did find a significant increased gene expression of WNT16 before nbUVB treatment in non-lesional skin compared with lesional skin in the population receiving nbUVB treatment. It is difficult to explain this discrepancy, as the two populations are comparable when it comes to number of participants, PASI and BMI. However, patients in the nbUVB treatment group were younger and included fewer female patients. Remarkably, our IHC results show an intense WNT16 protein expression in lesional skin compared to healthy controls and suggest increased expression in non-lesional skin compared to healthy controls. WNT16 has previously been shown to activate human keratinocyte proliferation, possibly via a β-catenin independent non-canonical WNT transduction pathway [46]. In the light of these results, it is tempting to assign WNT16 a key role in epidermal hyperproliferation but more studies are needed to determine if WNT16 is involved in the pathogenesis of psoriasis.

Our study found TCF7L2 gene expression to be lowered in lesional compared to non-lesional skin, with no significant difference in healthy controls. TCF7L2 has to our knowledge not been studied in patients with psoriasis before, but TCF7L2 has previously been connected to known comorbidities of psoriasis such as dyslipidemia, metabolic syndrome, Crohn’s disease and type 2 diabetes [19, 30, 41]. In Crohn’s disease, TCF7L2 gene is associated with decreased antimicrobial function of the Paneth cell where a diminished expression of TCF7L2 mediates a defective differentiation. This results in a deficiency in the antimicrobial shield, which enables the luminal microbes to invade the mucosa and cause inflammation. This is believed to be important in the pathogenesis of the disease [18, 29, 31]. Crohn’s disease and psoriasis have similarities in the immune response pattern and they share multiple genetic susceptibility loci [13, 39]. A defective differentiation of keratinocytes is characteristic of psoriasis [11], and it is speculated that specific bacteria in the skin of genetically predisposed individuals activate the innate immune system leading to inflammation [15]. It is interesting to speculate that the decreased gene expression of TCF7L2 in lesional skin found in our study contribute to these processes and that increased levels of TCF7L2 in lesional skin after nbUVB treatment play a role in normalizing the differentiation of the keratinocytes and restoring the antimicrobial host defence.

In this study, we also analyzed SNPs in WNT7B, WNT10B, WNT16 and TFC7L2 to investigate a possible association with psoriasis. WNT signaling, gene expression and genetic polymorphisms have previously been shown to be linked to several comorbidities associated with psoriasis. Several WNT10B SNPs have been associated with obesity [10, 26], and WNT10B has been implicated in regulating insulin sensitivity via skeletal muscle cells, leading to improved insulin sensitivity [1]. Genetic variants of TCF7L2 correlate with susceptibility to type 2 diabetes [8, 21] and Crohn’s disease [29, 30] and the aberrant expression of TCF7L2 has also been linked to the risk of cancer [7, 14]. Our results suggest that the analyzed SNPs in this study are not significantly associated with psoriasis, but this could be due to the relatively small size of our study population. It is possible that a larger study population would show different results.

There are other limitations to our study. Psoriasis patients analyzed for gene expression were significantly older and had a significantly higher body mass index (BMI) compared to the control group. However, there was no association between gene expression and age, PASI, or BMI in our data. Our results originate from a group of study participants with mild–moderate psoriasis with PASI < 10 and results might differ if patients with more severe disease had been included in the study. The conclusions that can be drawn from our IHC results are limited due to the small number of participants.

In conclusion, our results show for the first time a significantly decreased gene expression of WNT7B, WNT10B and TCF7L2 when comparing lesional and non-lesional skin in patients with psoriasis, and a significantly decreased gene expression of WNT7B and WNT10B when comparing lesional skin with healthy controls. NbUVB treatment significantly increases gene expression of WNT7B, WNT10B and TCF7L2 in lesional skin. Our IHC results show increased WNT16 protein expression in lesional skin compared to healthy controls. The functional contribution of WNT signaling to the pathophysiology of psoriasis needs to be studied further, but it can be speculated that WNT7B, WNT10B, TCF7L2 and WNT16 contribute to the pathogenesis of psoriasis.

## Electronic supplementary material

Below is the link to the electronic supplementary material.

Supplementary material 1 (PDF 103 kb)
