# Supplementary material for: Narrowband UVB treatment induces expression of WNT7B, WNT10B and TCF7L2 in psoriasis skin
Source: Arch Dermatol Res. 2019 May 14;311(7):535–44. doi: 10.1007/s00403-019-01931-y (PMC6677878; doi:10.1007/s00403-019-01931-y)
Supplement: Supplementary file 1 — Supplementary material 1 (PDF 103 kb) [file 403_2019_1931_MOESM1_ESM.pdf]

Narrowband UVB treatment induces expression of WNT7B, WNT10B and TCF7L2 in psoriasis skin

Archives of Dermatological Research

Malin Assarsson, Jan Söderman, Albert Duvetorp, Ulrich Mrowietz, Marita Skarstedt, Oliver Seifert*

*corresponding author
Oliver Seifert, MD, PhD
Division of Dermatology
Ryhov Hospital
S-55185 Jönköping, Sweden
E-mail: oliver.seifert@rjl.se

Gene	TaqMan Gene Expression Assay
WNT7B	Hs00536497_m1
WNT10B	Hs00559664_m1
WNT16	Hs00365138_m1
TCF7L2	Hs01009044_m1
TBP	Hs00427620_m1
ACTB	Hs99999903_m1
GAPDH	Hs03929097_g1

Supplementary table 1. TaqMan Gene Expression Assays (Applied Biosystems)

Gene	SNP	Assay Number
WNT7B	rs28578752	C_61267573_10
WNT7B	rs28439308	C_61267587_10
WNT7B	rs28663466	C_61267548_10
WNT10B	rs1051886	C_7470505_1
WNT10B	rs833843	C_7470507_20
WNT16	rs3779381	C_30135608_10
WNT16	rs2707466	C_2665840_20
WNT16	rs380138721	C_27518460_10
WNT16	rs2908004	C_15857691_10
TCF7L2	rs8766	C_8923408_10
TCF7L2	rs7901275	C_31976182_10

Supplementary table 2. TaqMan SNP genotyping assays (Applied Biosystems)

Psoriasis vs Controls					
	allele	frequency %	p-value	OR	95 % CI
WNT7B					
rs28663466	A	35.2 vs 32.2	0.33	1.14	0.87 - 1.50
rs28439308	C	32.9 vs 30.8	0.48	1.10	0.84 - 1.45
rs28578752	C	31.4 vs 27.8	0.22	1.19	0.90 - 1.57
WNT10B					
rs833843	G	59.4 vs 53.6	0.07	1.26	0.97 - 1.26
rs1051886	A	43.5 vs 36.8	0.03*	1.32	1.02 - 1.72
WNT16					
rs2707466	T	42.3 vs 43.4	0.74	0.96	0.74 - 1.24
rs2908004	A	42.0 vs 43.5	0.64	0.94	0.73 - 1.21
rs3779381	G	20.5 vs 23.4	0.29	0.84	0.62 - 1.16
rs3801387	G	21.7 vs 24.5	0.31	0.85	0.63 - 1.16
TCF7L2					
rs8766	C	45.8 vs 39.9	0.06	1.27	0.98 - 1.65
rs7901275	C	40.0 vs 44.5	0.16	0.83	0.64 - 1.08

Supplementary table 3 Allele frequencies of WNT7B, WNT10B, and WNT16 and TCF7L2 gene polymorphisms in patients with psoriasis (number of patients, n=170) and in healthy control subjects (n=365). Both case and control genotypes were in Hardy-Weinberg equilibrium (OR = odds ratio, CI = confidence interval, * not significant after correction for multiple testing).
